# Rapid Diagnosis of Intestinal Parasitic Protozoa, with a Focus on *Entamoeba histolytica*

**DOI:** 10.1155/2009/547090

**Published:** 2009-06-25

**Authors:** Anjana Singh, Eric Houpt, William A. Petri

**Affiliations:** ^1^University of Virginia, Charlottesville, P.O. Box 801340, VA 22908-1340, USA; ^2^Central Department of Microbiology, Tribhuvan University, Kirtipur, Kathmandu, Nepal; ^3^Infectious Diseases and International Health, University of Virginia, MR4 Building, Health System, Charlottesville, VA 22908-1340, USA

## Abstract

*Entamoeba histolytica* is an invasive intestinal pathogenic parasitic protozoan that causes amebiasis. It must be distinguished from *Entamoeba dispar* and *E. moshkovskii*, nonpathogenic commensal parasites of the human gut lumen that are morphologically identical to *E. histolytica*. Detection of specific *E. histolytica* antigens in stools is a fast, sensitive technique that should be considered as the method of choice. Stool real-time PCR is a highly sensitive and specific technique but its high cost make it unsuitable for use in endemic areas where there are economic constraints. Serology is an important component of the diagnosis of intestinal and especially extraintestinal amebiasis as it is a sensitive test that complements the detection of the parasite antigens or DNA. Circulating Gal/GalNac lectin antigens can be detected in the serum of patients with untreated amoebic liver abscess. On the horizon are multiplex real-time PCR assays which permit the identification of multiple enteropathogens with high sensitivity and specificity.

## 1. Introduction

The World Health Organization (WHO) ranks diarrheal disease as the second highest cause of morbidity and mortality in children in the developing world [1–3]. Enteric protozoa are one case of diarrheal disease in children. Intestinal protozoa are transmitted by the fecal-oral route and exhibit life cycles consisting of a cyst stage and a trophozoite stage. The cysts consist of a resistant wall and are excreted in the feces. The cyst wall functions to protect the organism from desiccation in the external environment. Unhygienic conditions promote transmission of most protozoa. Traditionally parasites have been identified by simple microscopy and serologic methods. New approaches include antigen detection and PCR [4–7].

## 2. Intestinal Parasites: *Cryptosporidium*, * Cyclospora, Entamoeba* spp., *Giardia, Isospora*

### 2.1. Cryptosporidium

The genus *Cryptosporidium * was identified in mice by Edward Tyzzer in 1907 [3]. It was found as human pathogen in 1976. Many species infect humans and a wide range of animals. *Cryptosporidium parvum * and *Cryptosporidium hominis * are the most prevalent species causing disease in humans [8]. Human cryptosporidiosis is also seen with *C. felis, C. meleagridis, C. canis, C. suis, * and *C. muris * [9–11]. In developing countries *Cryptosporium * spp. infections occur mostly in children younger than five years, with most under two years of age [12, 13]. *C. hominis * is the genus which infects only humans while *C. parvum * infects humans and cattle [11]. Recent literature shows that * C. hominis * is the commonest strain found in human stools [9, 14]. Each oocyst measures about 5.2 × 4.6 micrometers and contains four infective sporozoites. Recently *C. hominis * subgenotyping indicated that the infections included a wide range of subtypes consisting of three subtype families (Ia, Ib, and Id) [3].

### 2.2. Cyclospora

*Cyclospora cayetanensis * is a sporulating parasitic protozoan that infects the upper small intestinal tract. It has been identified as both a food and waterborne pathogen endemic in many developing countries. The disease first came to medical attention in the 1970s [15]. It is an important agent of Traveler's Diarrhea in developed countries and was responsible for numerous food borne outbreaks in the United States and Canada in the late 1990s. Approximately 1500 people during 1996 had *Cyclospora cayetanensis * diarrhea from Guatemalan raspberries. This epidemic recurred in 1997, emphasizing the risks of the global economy and food supply [16]. The ribosomal DNA of *C. cayetanensis * and three other species show a high degree of homology within each other. The *Cyclospora * homology and the lack of its sequence data from other species have hindered identification methods [17]. The incidence of infection of *Cyclospora * is high in the warmer months. Cyclosporiasis was found to be associated with ownership of domestic animals, especially birds, guinea pigs, and rabbits [18]. Many aspects of this disease and its transmission remain still an enigma.

### 2.3. Entamoeba spp.

#### 2.3.1. Entamoeba dispar

*E. dispar * exists in the colonic lumen as a harmless saprophyte [19]. *E. dispar * and *E. histolytica * are morphologically identical and phylogenetically closely related (~98% identity of rRNA sequences). Both species have a similar host range but have vastly different properties with regard to pathogenicity in vivo [20]. Both *E. histolytica * and *E. dispar * are able to colonize humans but only *E. histolytica * is able to cause invasive disease (colitis and extraintestinal manifestations). Tissue destruction is not seen with *E. dispar * in vivo. Earlier a panel of researchers concluded that colonization with *E. dispar * has never been documented to cause invasive disease in humans therefore the parasite does not necessitate treatment [21–23].

#### 2.3.2. Entamoeba histolytica

The main purpose of detection and differentiation of *E. histolytica * species in stool samples is the detection of the causative agent of amoebic dysentery. About 40–50 million people develop clinical amoebiasis each year, resulting on up to 100 000 deaths [24]. The causative agent of amebic colitis and liver abscess is *E. histolytica*. The non pathogenic parasites *E. dispar * and *E. moshkovskii * are more common and identical in appearance to *E. histolytica * [25, 26]. Invasive strains of *E. histolytica * may cause the deaths; the value (above) for the prevalence of *E. histolytica * is an overestimate since it dates from before the separation of the pathogen *E. histolytica * from the nonpathogen *E. dispar * [26]. Furthermore there are six additional species of amebae (*Entamoeba coli, Entamoeba hartmanni, Entamoeba polecki, Entamoeba chattoni, Iodamoeba butschlii * and * Endolimax nana*) that infect humans [27–37]. There are other amebae that infect humans, that is, Acanthamoeba that cause intestinal infections in humans. *E. nana * and *I. butschlii * colonize the human intestine and are nonpathogenic [38]. Infection due to *E. dispar * is 10 times more common than *E. histolytica *in developed countries [39–43]. Similarly even in a developing country *E. histolytica * and *E. dispar * can be equally prevalent [35]. *E. histolytica * and * E. dispar * share almost 90% genomic identity, and *E. moshkovskii * is also closely genetically related [44].

#### 2.3.3. Entamoeba moshkovskii

The free-living and parasitic amoeba * Entamoeba moshkovskii * is indistinguishable in its cyst and trophozoite forms from *E. histolytica * and *E. dispar*. *E. moshkovskii * has recently been shown to be a common infection of humans in the Indian subcontinent. Early isolates of *E. moshkovskii * were from sewage [45]. *E. moshkovskii * is osmotolerant and identified by growth at room temperature and by riboprinting [45–48]. Human isolates of *E. moshkovskii * have come from North America, South Africa, Bangladesh, and Italy [49, 50]. The pathogenic role of *E. moshkovskii * is yet to be defined. To minimize the confusion with *E. histolytica*/*E. dispar * a diagnostic tool is needed. *E. moshkovskii * prevalence suggests that the infection is common among children [50].

### 2.4. Giardia

*Giardia * is a binucleated flagellated protozoan and was discovered by Van Leeuwenhoek in 1681. Giardiasis is the most frequent cause of nonbacterial diarrhea throughout the world [51]. Each year 500 000 new cases are reported and about 200 million people develop symptomatic giardiasis [52]. These parasites can be found in mammals and other animals, including reptiles and birds. *Giardia lamblia * (syn. *duodenalis * or *intestinalis*) has two anterior nuclei of equal size that contain complete copies of the genome [53]. The parasite has a ventral adhesive disc made of microtubules. There are four pairs of flagella (one anterior pair, two posterior pairs) and a caudal pair that emerges from the disc. The complex working of the unique *Giardia * cytoskeleton has been reviewed [54]. *Giardia * cysts are resistant to chlorination and ozonolysis and can remain viable for several weeks, especially in cold surface water. The acquisition of *Giardia * occurs most commonly through ingestion of the cyst in contaminated water or food. Even flies can spread viable *Giardia lamblia * cysts on their exoskeleton, which they have acquired naturally from unhygienic sources [55]. There are two distinct genotypes of *G. lamblia * that infect humans, commonly referred to as assemblages A and B. Molecular analyses have shown the genetic variance between the two assemblages to be greater than that used to delineate other species of protozoa [56]. Furthermore, it has been hypothesized that there may be phenotypic differences between assemblages. One study showed an association between intermittent diarrhoea and assemblage A and between persistent diarrhoea and assemblage B [57]. Others studies showed that children with assemblage A were more likely to be symptomatic [58]. A recent study showed that the majority of *G. lamblia * infections in a northeastern Brazilian community were assemblage B [59].

### 2.5. Isospora

Isosporiasis is a human intestinal disease caused by the parasite *Isospora belli*. It is found worldwide, especially in tropical and subtropical areas. It was first documented in 1915. Infection is seen most frequently in immunocompromised individuals. *I. belli * is a coccidian protozoa in phylum Apicomplexa that parasitizes epithelium of upper small intestine of humans and causes diarrheal disease. The entire life cycle of *Isospora * consists of asexual development and sexual reproduction that take place in the same host. Transmission of *I. belli * oocysts seems to be confined to the anthroponotic cycle because humans are the only known natural host [60]. The oocysts of *I. belli * usually require less than one day to a few days to complete sporogonic development and become infective [61, 62].

## 3. Methodological Approaches, with a Focus on Amebiasis Diagnostics

### 3.1. Microscopic

For amebiasis, microscopy cannot distinguish *E. histolytica * from the more common parasites *E. dispar * and *E. moshkovskii. * It is therefore an obsolete approach to the diagnosis of amebiasis, but still conducted in most parts of the world where modern diagnostic approaches have failed to take hold. For microscopy each stool sample should be divided into two portions. Direct microscopy should be done by mixing a small amount of the specimen in 0.9% sodium chloride solution (wet mount) or Lugol's iodine solution. This allows the detection of motile trophozoites of *Entamoeba histolytica/dispar * and can also provide information on the contents of the stool, that is, the presence of leucocytes and red blood cells. The second portion of the stool sample is then stained with trichrome and/or iodine to identify trophozoites and cysts. Three negative stool samples are required before it can be accepted to report that there is no amoebic infection [63]. Trophozoites containing ingested RBCs are more common with *E. histolytica * than *E. dipar * [64–66]. The sensitivity of microscopy is as less as 60% and confounded with misleading results due to misidentification of macrophages as trophozoites, (polymorphonuclear leukocytes) PMNs as cysts (particularly when lobed nuclei of PMNs break apart), and other “*Entamoeba * species” [64, 66–70].

### 3.2. Serology

The combination of serology and stool antigen assays is more sensitive and specific than microscopy for the diagnosis of *Entamoeba histolytica * infection [42]. The tests of choice for serology are indirect fluorescent antibody test (IFAT), counter immunoelectrophoresis (CIEP), or enzyme linked immunosorbent assay (ELISA). Serologic tests are positive at the time of clinical presentation of amebiasis in 60–90% of cases, with positive serology seen in the overall population of endemic areas of 5–10% (raising the issue of both false positive and false negative results with serology).

#### 3.2.1. Dipstick

Point of care tests to detect amebiasis would be appropriate technology for the developing world. There are at least two such tests that are in the early stages of development [71, 72].

#### 3.2.2. Rapid Antigen Detection

Stool oocyst and parasites (O&Ps) exam cannot distinguish morphologically the three closely related common amoebae: pathogenic *E. histolytica * and commensal *E. dispar * and *E. moshkovskii*. Differentiation of *E. histolytica * from *E. dispar * most practically can be accomplished by antigen detection. Currently there are several antigen detection tests commercially available for in vitro diagnostic use. The TechLab *E. histolytica * II test detects exclusively *E. histolytica * [73, 74]. Commercial enzyme-linked immunosorbent assays from Merlin and Alexon do not differentiate between *E. histolytica * and *E. dispar * [75, 76]. Buss et al. concluded that the two ELISAs used in their study were relatively quick and easy to perform but the Techlab *E. histolytica * II ELISA outperformed the R-Biopharm Ridascreen *Entamoeba * test [77]. Sensitivity and specificity of the TechLab kit have been studied from all over the world viz. Bangladesh [73], Canada [42], the Netherlands [78], the United Kingdom [79], and India [80]. 

*E. histolytica * infection can also be detected through Gal/GalNAc lectin antigen in serum. The advantage is that it is a more sensitive method than detection of antilectin antibody for the early diagnosis of amebic liver abscess (ALA). It is also more specific and uses a well-defined antigen, the Gal/GalNAc lectin. It can also, unlike antibody detection tests, be used as a test of treatment [81]. A disadvantage of this method is that the sensitivity of this method is significantly decreased in ALA patients after initiation of antiamoebic therapy. 

Salivary antigen has also been tested as a predictor for invasive disease. In one of the studies it was found that the presence of lectin in saliva had moderate sensitivity (65.8%) and high specificity (97.4%) in early infections (<1 week amebic colitis). Although the noninvasive sample collection is an advantage, the sensitivity of this assay appears to be lower than that of serum antigen detection [82].

### 3.3. PCR

#### 3.3.1. Conventional PCR

Diagnosis of *E. histolytica * by PCR tests started in the early 1990s. Differentiation of *E. histolytica * from *E. dispar * by restriction fragment analysis of a single gene amplified in vitro was first reported in 1991 [83]. PCR-based approaches have been endorsed by the WHO, and in developed countries has found application in clinical and epidemiological studies [84–87]. Identification of *E. histolytica * can be done from various clinical specimens, such as stool, tissues, and liver abscess aspirate [70]. Though PCR of 18S rDNA is expensive, it is as sensitive as ELISA techniques [88–93]. PCR methods were found to be highly sensitive and specific for detecting parasite DNA from microscopy-positive samples using both manual and automated methods [94–101]. PCR assays targeting 18S rDNA are widely used for the detection and differentiation of *Entamoeba * species. This can be easily detected from a DNA fragment of a single-copy gene or from multicopy, extrachromosomal plasmids in the amoebae [102]. Amplification of *E. histolytica * and *E. dispar * DNA fragments from human stool by conventional PCR has been established to be a sensitive and specific method for its detection [100]. Extraction of DNA was performed directly from stool and amplified using primers that amplify the extra chromosomal circular DNA [100, 101, 103]. Microscopically positive *E. histolytica * positive clinical 27/30 (90%) fecal specimens and 3/30 (10%) liver abscess aspirates from Pharmongkutklao and Ramathibodi hospitals in Bangkok, Thailand were evaluated by PCR. All specimens were reported as positive for *Entamoeba * cysts or trophozoites by microscopic examination. After being tested with a genus-specific PCR assay [35], 25/30 (83%) samples were positive for *Entamoeba * spp. whereas 5/30 (16.6%) samples were negative. By using the PCR assay developed successfully identified 10/30 (33.3%) clinical samples tested: 4/10 (40%) was positive for *E. histolytica * 6/10 (60%) for *E. dispar. * The same results were obtained when previously described *E. histolytica-*specific and *E. dispar-*specific primers were used [104]. No amplification of *E. moshkovskii * was observed with any specimens [85].

For the simultaneous detection and differentiation of *E. histolytica * and *E. dispar * from DNA extracted from microscopy-positive fecal samples (fresh and formalin-fixed) multiplex PCR was developed with a reported sensitivity and specificity of 94% and 100%, respectively [86, 105, 106]. Haque et al. identified *E. moshkovskii * in fecal specimens using a riboprinting method [49]. A PCR test for the identification of *E. moshkovskii * in fecal samples was developed and shown to have a high sensitivity and specificity using DNA extracted directly from stool samples with the QIAGEN stool extraction kit [106, 107]. A simpler PCR molecular detection tool developed by Ali et al. for diagnosing *E. moshkovskii * infections was used to detect the parasite directly in stool. Out of 109 tested stool specimens from preschool children in Bangladesh by PCR, *E. histolytica * was detected in 17/109 (15.6%), *E. dispar * in 39/109 (35.8%), *E. moshkovskii * in 23/109 (21.1%), mixed infection of *E. histolytica * and *E. dispar * in 17 (73.9%), and *E. dispar * and *E. moshkovskii * coinfection in 11/23 (48%) [50]. The high association of *E. moshkovskii * with * E. dispar * may have obscured its identification in previous studies.

#### 3.3.2. Real-Time PCR

The beauty of a newly developed real-time PCR (qPCR) methodology for laboratory diagnosis of infectious diseases is that it is more sensitive than conventional PCR, is more rapid, leading to shorter turnaround times, has a reduced risk of amplicon contamination from laboratory environments, and has reduced reagent costs [108]. Specific detection of the amplicon occurs, enabling continuous monitoring of amplicon (PCR product) formation throughout the reaction. In comparison to conventional PCR, real-time PCR is more sensitive and is also quantitative. Several qPCR methods have been designed [109–111]. Clinical specimens may contain impurities that might inhibit enzyme-based nucleic acid amplification. Therefore, the use of internal controls (ICs) for the routine diagnostic PCR provides assurance that the clinical specimens are successfully amplified and detected. 

For single-plex real-time PCR detection of *E. histolytica, * Qvarnstrom et al. used TaqMan probes targeting the 18S rRNA gene, with the SYBR Green approach offering a good alternative (but not sequence-specific) to the TaqMan assay [109].

Verweij et al. developed a multiplex qPCR assay for detection of three different intestinal parasites *E. histolytica*, *G. lamblia*, and *C. parvum*. Their study showed 100% (20/20) amplification of *E. histolytica * and *G. lamblia * DNA in microscopically positive isolates. Further, in 20 samples in which modified acid-fast staining revealed *Cryptosporidium * oocysts and in 4/7 (57%) samples from an immunocompromised child with complaints of diarrhea, *C. parvum * DNA was detected with the qPCR tested [111]. Verweij et al. showed multiplex PCR 100% specificity and sensitivity for *E. histolytica * and *G. lamblia, * and *C. parvum * [112]*.*

Later Haque et al. produced a multiplex real-time PCR assay for the detection of *E. histolytica*, *G. lamblia*, and *C. parvum*. The detection limit for the multiplex real-time PCR was 1 trophozoite of *E. histolytica * per extraction (100 *μ*L), 10 trophozoites of *G. intestinalis * per extraction, and 100 oocysts of *Cryptosporidium * per extraction [21]. The multiplex qPCR assay demonstrated 83/97 (85%) agreement with microscopy for *Giardia*, with specificity for *E. histolytica * and *G. lamblia, * and *C. parvum * of 98%, 97%, and 100%, respectively [21].

In another study qPCR for *E. histolytica * was positive in 20/23 (87%) liver abscess pus specimens, with the 3 negative specimens from samples collected from patients who had already received antiamebic therapy [108]. Results have been highly specific and sensitive [40]. 

Stroup et al. developed a *Cryptosporidium * qPCR species-specific probe assay that is sensitive and simple to perform. The assay was done on 123 human stool specimens from Bangladesh and Tanzania and exhibited a sensitivity and specificity of >91% versus microscopy. *Cryptosporidium parvum-*specific and *Cryptosporidium meleagridis *-specific scorpion qPCR assays provided 100% accurate speciation compared with Vspl RFLP analysis and sequencing [113].

An *Isospora belli * qPCR assay was performed with 21 positive and 120 negative stool samples and achieved 100% specificity and sensitivity. PCR could supplement the clinical laboratory diagnosis of isosporiasis, in particular in patients with a history of diarrhea developing during or immediately after travel to developing countries [114].

## 4. Future Approaches

The burden of enteric protozoan infections is so great in developed and developing countries that there is a need for better diagnostic tests. The production of point-of-care lateral flow “dipstick” antigen detection tests and high-throughput screening tests based on antigen detection or PCR are clear priorities.

## 5. Conclusions

In the clinical laboratories the diagnosis of intestinal amebiasis should use a combination of detection of the parasite by antigen detection or PCR (using *E. histolytica * specific tests) and serological testing, and/or by colonoscopy and biopsy of intestinal amebic lesions, and in the case of amebic liver abscess by a combination of serology and drainage of the liver abscess with testing of the fluid for the parasite ideally by PCR. The development of molecular tools, including antigen detection and PCR and qPCR, to detect *E. histolytica, E. dispar*, *E. moshkovskii*, *Giardia * spp, and *Cryptosporidium* spp. DNA in stool or liver abscess samples promises to provide major advances. The amalgamation of many new technologies into the diagnostic laboratory will represent a challenge to all, but may lead to a better understanding of the public health problems represented by these diseases.
